# Sexual Dimorphism in Offspring Glucose-Sensitive Hypothalamic Gene Expression and Physiological Responses to Maternal High-Fat Diet Feeding

**DOI:** 10.1210/en.2014-1131

**Published:** 2014-03-31

**Authors:** Laura Dearden, Nina Balthasar

**Affiliations:** School of Physiology and Pharmacology, University of Bristol, Bristol BS8 1TD, United Kingdom

## Abstract

A wealth of animal and human studies demonstrate that early life environment significantly influences adult metabolic balance, however the etiology for offspring metabolic misprogramming remains incompletely understood. Here, we determine the effect of maternal diet per se on offspring sex-specific outcomes in metabolic health and hypothalamic transcriptome regulation in mice. Furthermore, to define developmental periods of maternal diet misprogramming aspects of offspring metabolic balance, we investigated offspring physiological and transcriptomic consequences of maternal high-fat/high-sugar diet feeding during pregnancy and/or lactation. We demonstrate that female offspring of high-fat/high-sugar diet-fed dams are particularly vulnerable to metabolic perturbation with body weight increases due to postnatal processes, whereas in utero effects of the diet ultimately lead to glucose homeostasis dysregulation. Furthermore, glucose- and maternal-diet sensitive gene expression modulation in the paraventricular hypothalamus is strikingly sexually dimorphic. In summary, we uncover female-specific, maternal diet-mediated in utero misprogramming of offspring glucose homeostasis and a striking sexual dimorphism in glucose- and maternal diet-sensitive paraventricular hypothalamus gene expression adjustment. Notably, female offspring metabolic vulnerability to maternal high-fat/high-sugar diet propagates a vicious cycle of obesity and type 2 diabetes in subsequent generations.

A worldwide increase in the overweight and obese population is driving an intense research focus on the identification of pathways leading to excessive weight gain and its accompanying pathologies. A key aspect of this research is an assessment of the contribution of hereditary influences, such as genetic variations underpinning an increased susceptibility to weight gain (1–4). In addition to these genetic factors, of which many remain to be identified and characterized, a wealth of animal and human studies have provided evidence that early life environment significantly influences adult metabolic balance. A solid body of evidence shows that offspring of obese mothers, be it rodents, monkeys, or humans (5–7), are programmed to become overweight themselves, in part by epigenetic mechanisms changing gene expression into adulthood.

Over the past two decades the important roles the hypothalamus plays in the regulation of whole body energy and glucose homeostasis have become increasingly clear (8). Appropriate nutrient and hormone exposure of the fetus in utero as well as during early postnatal periods is key to healthy development and regulation of these hypothalamic pathways (9–11). Although it has been debated whether maternal obesity itself or the quantity/quality of maternal diet misprograms offspring metabolic balance, recent primate data suggest that maternal high-fat diet (HFD) feeding causes placental dysfunction and metabolic perturbation in offspring independent of maternal obesity (6, 12). However, at which developmental time point maternal diet as such might influence aspects of offspring metabolic health remains debated. Important aspects to consider are composition of the diet, as well as sex-specific vulnerabilities to maternal diet. Because there is limited data to suggest that genders may respond differently to maternal diet [for review see Aiken and Ozanne (13)], most developmental programming studies include only one sex. Furthermore, sexually dimorphic offspring responses to maternal diet have not been addressed in the CNS.

In addition to body weight increases, disturbance in the maintenance of glucose homeostasis has been reported in offspring of obese and HFD-fed mothers (14, 15), however at which developmental stage offspring glucose homeostasis dysregulation is programmed by maternal diet or maternal metabolic state is currently unclear. Hypothalamic neurons are exquisitely sensitive to changes in extracellular glucose-levels (16) and disturbance of their ability to respond appropriately leads to dysfunctional maintenance of whole body glucose homeostasis (17). HFD-induced obesity is one of the conditions leading to dysfunctional hypothalamic glucose-sensing (17), however, whether maternal diet affects offspring glucose-sensitive mechanisms is unknown.

The current study thus aimed to determine the effect of maternal diet per se (rather than maternal overweight) on offspring sex-specific outcomes in metabolic health and specifically glucose homeostasis, as well as identifying the developmentally critical time periods of maternal diet affecting particular aspects of metabolic balance. We have previously described hypothalamic mechanisms linking glucose-sensing to appropriate transcriptome regulation (18), and we thus aimed to investigate whether offspring hypothalamic glucose-sensitive gene expression may be altered by maternal diet.

## Materials and Methods

Studies were carried out in accordance with the UK Animals (Scientific Procedures) Act 1986 and with the approval of the local ethical committee. CD1 mice were obtained from B&K Universal Ltd. Mice were maintained on a 12-hour light/dark cycle with free access to water and chow diet unless otherwise stated (EURodent Diet 22%; LabDiet).

### Maternal programming studies

Virgin mice were mated at 8 weeks of age and pregnancy detected by the presence of a semen plug. Plugged females were single caged and randomly allocated to either a chow or HFD (D12331, Research Diets Inc; 58% fat [hydrogenated coconut oil] plus high sucrose [25% carbohydrate]) for the duration of pregnancy and/or lactation. Litter size was normalized to ten pups per litter at P4. Offspring were weaned from mothers at P21 and all offspring were placed onto a chow diet.

### Weight, food intake, and ambient glucose measurements

Weight and blood glucose measurements were collected from dams every two days between 10:00 and 11:00 AM. Circulating blood glucose measurements were collected from a tail tip using a One Touch Ultra handheld glucose meter (Lifescan, Inc). Body weight and blood glucose measurements were collected from offspring either once every four days or once a week, depending on the study, in the same manner as for dams. Food intake was calculated every day (offspring) or once every four days (dams) at 10:00 AM from preweighed food portions dispensed from the food hopper. Food intake measurements represent the average 24-hour food intake calculated over the stated time period.

### Fasting glucose

Mice were single caged and fasted for 6 hours with access to water. Blood glucose measurements were collected by tail tip at the start and end of the fast.

### Glucose tolerance test

Mice were fasted for 6 hours and injected intraperitoneally (IP) with a 20% (wt/vol) glucose solution at 2g/kg body weight. Blood glucose levels were measured before and 15, 30, 45, 60, 90, and 120 minutes after glucose injection. For measurement of insulin release in response to glucose, animals were killed at 0, 30, or 60 minutes after glucose injection and truncal blood collected for further analysis.

### Insulin tolerance test

Mice were injected IP with insulin at 1.5 U/kg body weight. Blood glucose levels were measured before and 15, 30, 45, 60, 75, and 90 minutes after insulin injection.

### Adiposity measurements

Mice were terminally anesthetized and scanned using a Lunar Piximus DXA scanner analyzed with Lunar Piximus 2.10 software.

### Serum insulin levels

Truncal blood was collected from terminally anesthetized mice. Blood samples were centrifuged for 15 minutes at 3000 *g* and plasma collected. Plasma samples were analyzed using the Ultrasensitive Mouse Insulin ELISA kit (Crystal Chem).

### Insulin-induced hypoglycemia

Food was removed and animals were injected IP with human insulin (Humulin S, Eli Lilly and Company) at 2.5 U/kg body weight. Controls received a saline injection. After 1 hour, blood glucose levels were ascertained to be below 2.5 mM and animals culled by overdose of anesthetic (Euthatal).

### cDNA generation and real-time PCR

Microdissection of the paraventricular hypothalamus (PVH) and arcuate nucleus (ARC) samples was as previously described (19). After microdissection of the PVH and ARC from whole brains, RNA was extracted using Trizol according to manufacturers instructions (Invitrogen). RNA was DNAse treated (Ambion Inc) and reverse transcribed (Revert-aid, Fermentas). *Irs2*, *Crh, Kir6.2,* and *Glut3* mRNA was measured using TaqMan assay-on-demand (*Irs2*-Ro01482270_s1, *Crh*-Mm01293920_s1, *Kir6.2*-Mm00440050_s1, and *Glut3*-Mm00441483_m1; Applied Biosystems) and normalized to 18S rRNA (Applied Biosystems) using multiplexing on a Stratagene Mx3000P system (Stratagene).

## Results

### Glucose-sensitive hypothalamic gene expression alteration

**Figure 1.**
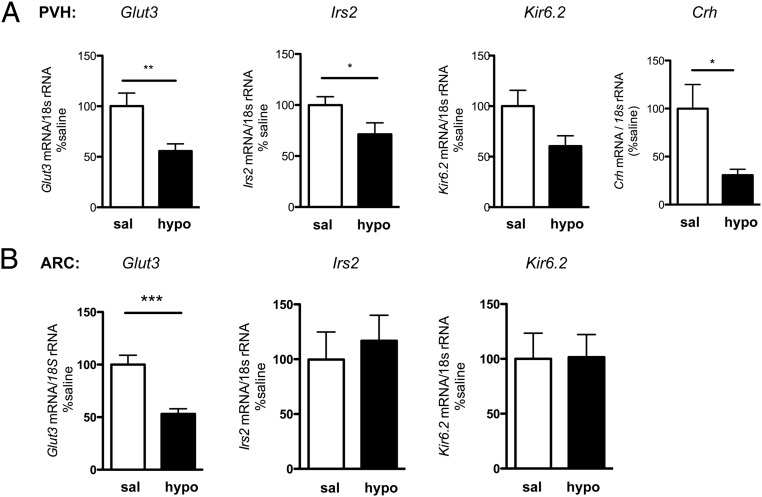
Glucose-sensitive hypothalamic gene expression modulation. A, PVH. B, ARC *Glut3, Irs2, Crh,* and *Kir6.2* mRNA expression (TaqMan probe and multiplexing against *18S* rRNA) was measured in male CD1 mice 60 minutes after either a saline injection (sal) or insulin-induced hypoglycemia (<2.5 mM blood glucose; hypo) (n = 5–9; *, *P* < .05; **, *P* < .01; ***, *P* < .001 *t*-test for individual genes).

Initially the modulation by insulin-induced hypoglycemia of candidate hypothalamic genes previously shown to be regulated by metabolic/glycemic state and/or implicated in the neuronal mechanisms sensing and responding to extracellular glucose levels (18, 20–22) was investigated in two glucose-sensitive hypothalamic areas, PVH and ARC, in adult male CD1 mice. A strong response to glycemic state was apparent, particularly in the PVH: expression of glucose transporter 3 (*Glut3*), insulin receptor substrate 2 (*Irs2*), the K_ATP_-channel subunit *Kir6.2,* and corticotrophin releasing hormone (*Crh*) were down-regulated during hypoglycemia 60 minutes after insulin injection (Figure 1A), suggesting rapid glucose-sensitive PVH transcriptome regulation. Glucose-sensitive gene expression regulation was also apparent in the ARC, but to a lesser degree (Figure 1B).

### A mouse model of maternal dietary programming

**Figure 2.**
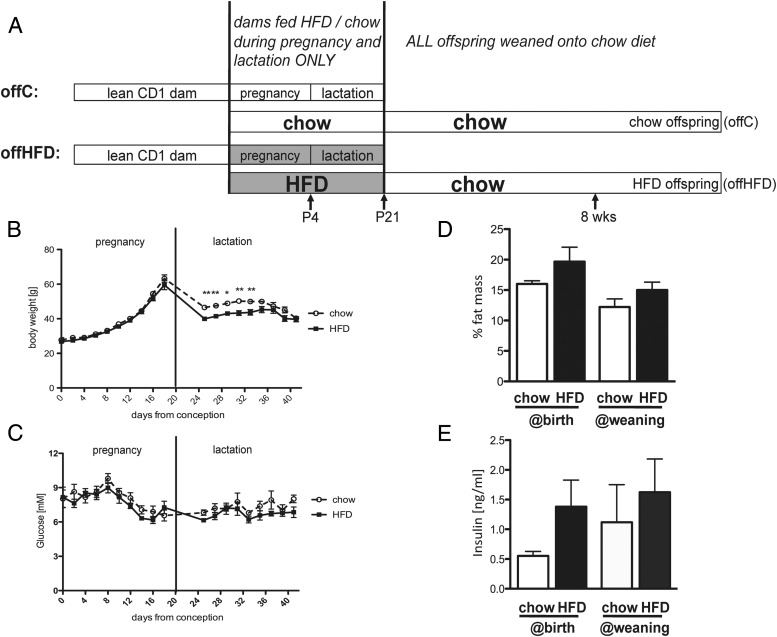
Mouse model of maternal dietary programming. A, Lean CD1 dams are fed either chow or a high-fat diet (HFD, 58% fat + high sucrose) during pregnancy and lactation only. All offspring is weaned onto chow and offspring from chow-fed dams (offC) compared with offspring from HFD-fed dams (offHFD) at P4, P21/weaning and at 8 weeks. B, Body weight and C, Blood glucose of chow- and HFD-fed dams during pregnancy and lactation (n = 8; **, *P* < .01; *, *P* < .05, 2-way repeated measures ANOVA). D, Dam adiposity was measured as %fat by DXA scanning a day after birth and at weaning (n = 4–5). E, Dam serum insulin was measured one day after birth and at weaning (n = 4–5).

To investigate whether maternal diet (rather than maternal overweight) might have consequences for offspring glucose-sensitive gene expression and/or whole body glucose homeostasis, lean CD1 dams were fed either chow or a high-fat/high-sucrose diet (58% fat + high sucrose; D12331 Research Diets) during pregnancy (from day of plug) and lactation only (Figure 2A). Outbred CD1 mice were chosen, as they are relatively resistant to HFD-mediated weight gain and their increased genetic variability more closely resembles human genetic variability. All offspring were weaned onto chow and offspring from chow-fed dams (offC) compared to offspring from HFD-fed dams (offHFD) at P4, P21/weaning, and at 8 weeks. Dams fed a HFD during pregnancy and lactation did not gain significant weight or develop hyperglycemia during this period (Figure 2, B and C). During the lactation period dams fed a HFD in fact temporarily lost more weight than chow-fed dams (Figure 2B). Calorie intake during pregnancy was the same in chow and HFD-fed dams (Supplemental Figure 1A). However, HFD-fed dams initially failed to increase food intake during lactation to meet increased energy demand from suckling pups (Supplemental Figure 1A), which might explain their temporary decrease in body weight during early lactation. Adiposity (measured as %fat by DXA scanning) was not significantly altered during pregnancy or lactation (Figure 2D), neither were serum insulin levels (Figure 2E) or insulin tolerance (Supplemental Figure 1B). HFD-feeding of pregnant dams did not affect litter size or offspring sex ratio (supplemental Figure 2). Data presented in Figure 2 are of one example cohort (n = 8 dams/diet), but are identical to 5 repeat cohorts of the same size. These data suggest that any observed offspring metabolic effects are due to maternal diet per se, rather than maternal overweight or hyperglycemia/insulin resistance.

### Female offspring of HFD-fed dams is more vulnerable to metabolic disturbance than males

**Figure 3.**
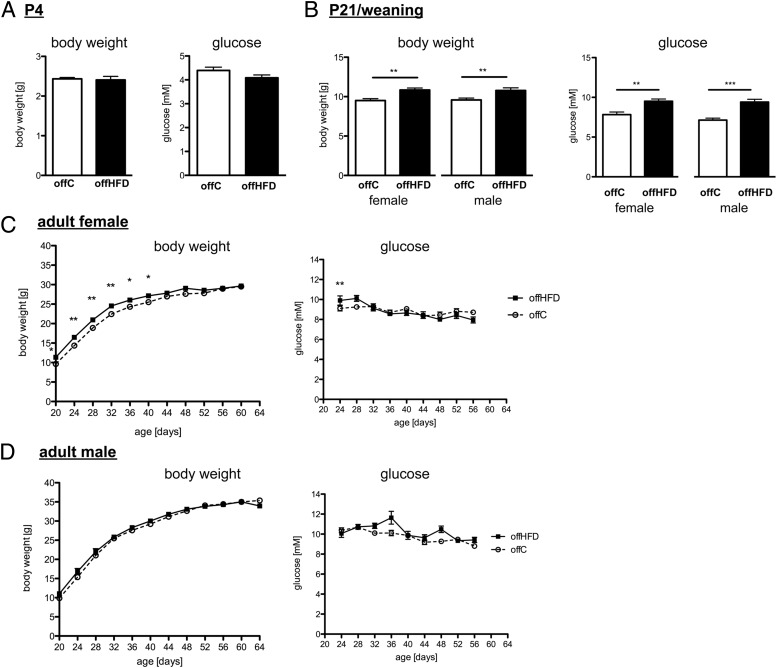
Female offspring of HFD-fed dams are more vulnerable to increased weight. A, Body weight and blood glucose of (unsexed) P4 offspring from chow-fed (offC) or HFD-fed (offHFD) dams (n = 15). B, Female and male offC and offHFD body weight and blood glucose at P21/weaning (n = 33–42; **, *P* < .01; ***, *P* < .001; 1-way ANOVA). C, Female body weight and blood glucose curves after weaning until 8 weeks of age. D, Male body weight and blood glucose curves after weaning until 8 weeks of age (n = 14–24; **, *P* < .01; *, *P* < .05; 2-way repeated measures ANOVA).

At P4 no difference in body weight or blood glucose was noted between unsexed offC and offHFD (Figure 3A). At P21 both female and male offHFD pups had significantly increased weight and blood glucose (Figure 3B). Female offHFD maintained increased weight for a further 3 weeks, whereas blood glucose levels remained increased only for a week post weaning (Figure 3C). Male offHFD body weight normalized within a week of weaning; blood glucose levels also returned to normal within 3 days (Figure 3D). Data presented here are representative of one study cohort, however the exact same protocol was repeated in 5 more cohorts of equivalent size and the exact same phenotype of offspring was found in every cohort.

**Figure 4.**
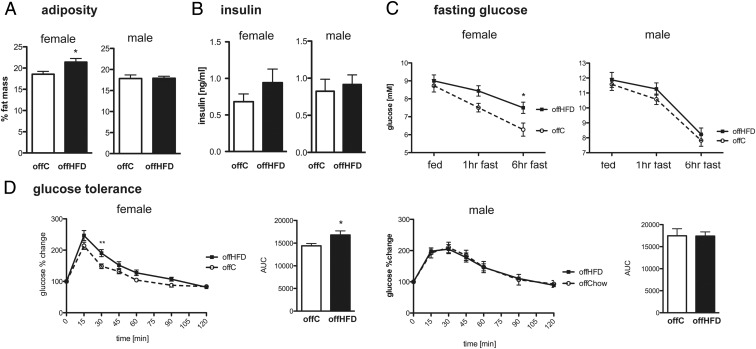
Adult female offspring of HFD-fed dams have dysfunctional glucose homeostasis. A, 8-week-old female and male offspring adiposity was measured as %fat by DXA scanning (n = 18–26; *, *P* < .05; *t*-test). B, Serum insulin levels of fed 8-week-old female and male offC and offHFD (n = 6–9). C, 8-week-old female and male offC and offHFD were fasted at 9:00 AM for 6 hours and blood glucose measured at 1 and 6 hours (n = 13–15; *, *P* < .05; 2-way repeated measures ANOVA). D, Glucose tolerance test (2g/kg) in 8-week-old female and male offC and offHFD (n = 8–11; **, *P* < .01; 2-way repeated measures ANOVA). Adjacent area under curve analysis (n = 8–11; *, *P* < .05; *t* test).

At 8 weeks of age, female mice, despite similar body weight (offC:29.2 ± 0.4g vs. offHFD:30.0 ± 0.4g, n = 18–21), had increased adiposity (Figure 4A), although serum insulin levels and insulin tolerance were not significantly altered (Figure 4B and Supplemental Figure 3A). Male offspring displayed no body weight (offC: 38.0 ± 0.5g vs offHFD 39.1 ± 0.5g, n = 21–26), adiposity (Figure 4A), insulin (Figure 4B), or insulin tolerance (Supplemental Figure 3A) phenotype. Lean mass was not affected by maternal diet for either sex (data not shown), nor was offspring food intake (Supplemental Figure 3C).

Female offHFD remained significantly hyperglycemic in the face of a 6-hour fast in comparison to offC (Figure 4C) and showed significant glucose intolerance (Figure 4D), whereas male offHFD mice had normal responses to either challenge (Figure 4, C and D). Furthermore, female offHFD glucose levels were higher 30 minutes after insulin injection, despite increased secretion of insulin at this time point compared with offC littermates (Supplemental Figure 3B), suggesting a mild decrease in insulin sensitivity. Male offHFD insulin responses to the glucose tolerance test are comparable to offC (Supplemental Figure 3B).

### Glucose- and maternal diet-mediated PVH gene expression regulation is sexually dimorphic

To assess whether maternal diet may affect glucose-sensitive PVH transcriptome regulation, PVH expression of genes previously found to be regulated by hypoglycemia (Figure 1) was investigated in offC and offHFD of both sexes.

At weaning, despite identical overweight and hyperglycemia phenotypes in male and female offHFD, PVH-specific expression of, for example, *Irs2* mRNA showed sexually dimorphic expression patterns; *Irs2* mRNA expression was unaffected by maternal diet in female offHFD, whereas it was significantly increased in male offHFD littermates (Supplemental Figure 4).

**Figure 5.**
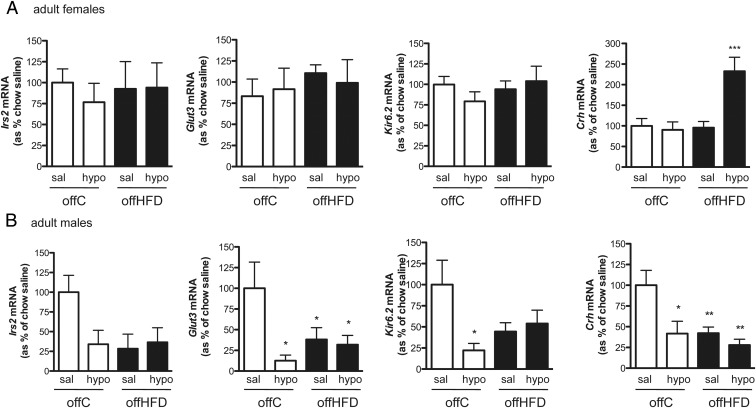
Sexual dimorphism in PVH glucose- and maternal-diet-sensitive gene expression regulation. cDNA was generated from microdissected PVH in 8-week-old A, female and B, male offC and offHFD mice 60 minutes after either a saline (sal) or 2.5U/kg insulin (hypo) injection and expression of *Irs2, Glut3*, *Kir6.2*, and *Crh* measured using TaqMan probes by multiplexing against *18S* rRNA (n = 6–11; **, *P* < .01; *, *P* < .05; 1-way ANOVA for individual genes)

This pattern continued at 8 weeks of age, where in contrast to male offspring, female PVH gene expression remained unaltered by maternal diet (offC sal vs offHFD sal; Figure 5A). In male offspring candidate genes were down-regulated by maternal diet (offC sal vs offHFD sal; Figure 5B). No gene regulation was observed in response to hypoglycemia in female offHFD, except for a significant up-regulation of *Crh* in response to hypoglycemia in offHFD mice (offHFD sal vs hypo, Figure 5A).

**Figure 6.**
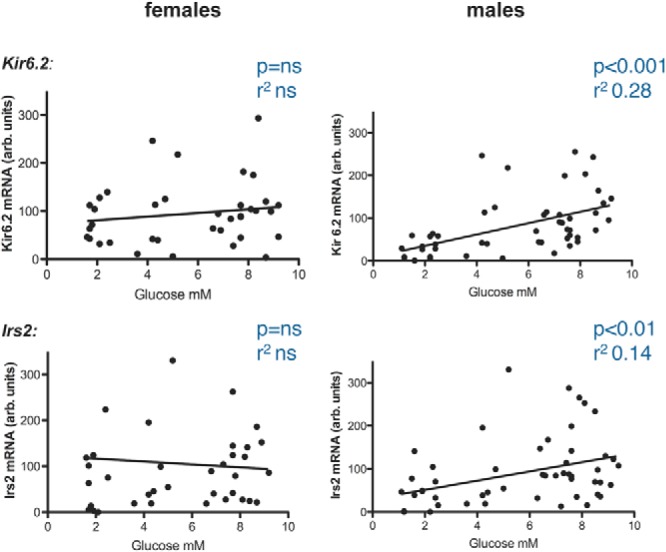
Absence of glucose:PVH gene expression correlation in female mice. *Kir6.2* (top panels) and *Irs2* mRNA (bottom panels) expression at different glycemic states in adult female (left panels) and male (right panels), normo-, hyper-, and hypoglycemic offC mice. Gene expression values derive from different experiments and are normalized to each experimental average (arb units).

As previously observed in male CD1 mice (Figure 1), male offC PVH *Irs2, Glut3*, *Kir6.2,* and *Crh* mRNA expression was down-regulated in response to insulin-induced hypoglycemia (offC sal vs offC hypo); however, no such change occurred in female offC, despite identical states of severe hypoglycemia in all groups (<2.5 mM, Figure 5, A and B). To further analyze this sexual dimorphism in glucose-responsive PVH gene expression, PVH gene expression was plotted against blood glucose levels for all adult male and female offC mice (Figure 6). Although significant correlations between glycemic state and PVH gene expression were observed in male mice, no such correlation indicating glucose-sensitive gene expression exists in female mice (Figure 6).

### In utero programming of offspring glucose homeostasis

**Figure 7.**
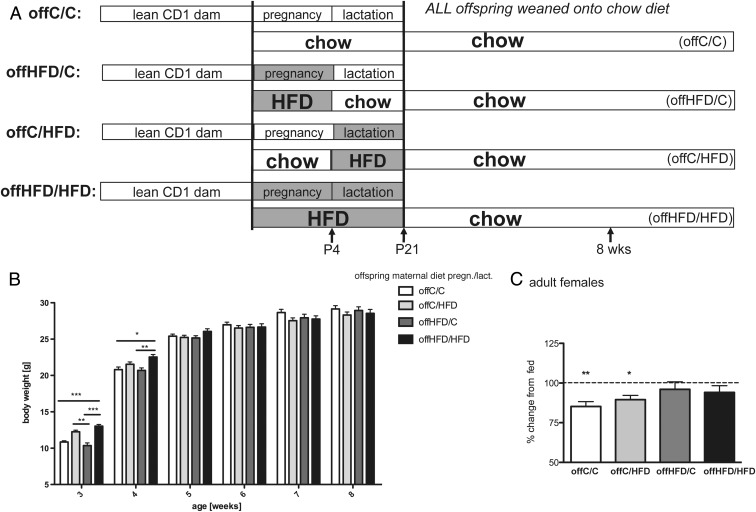
Glucose homeostasis dysregulation in female offspring of HFD-fed dams is programmed in utero. A, Lean CD1 dams (n = 6–8) are fed: chow during pregnancy and lactation (offspring is offC/C), HFD during pregnancy only (offspring is offHFD/C), HFD during lactation only (offspring is offC/HFD), or HFD during pregnancy and lactation (offspring is offHFD/HFD). All offspring is weaned onto chow. B, Offspring body weight (n = 18–26; ***, *P* < .001; **, *P* < .01; *, *P* < .05; 2-way repeated measures ANOVA). C, 8-week-old female offC/C, offHFD/C, offC/HFD, and offHFD/HFD were fasted at 9:00 AM for 6 hours. Blood glucose drop is expressed as %prefast (n = 10–14; **, *P* < .01; *, *P* < .05; 2-way repeated measures ANOVA).

To investigate the critical developmental period during which maternal diet programs offspring glucose homeostasis, dams were fed a HFD either during pregnancy only (offHFD/C), lactation only (offC/HFD) or during both pregnancy and lactation (offHFD/HFD) (Figure 7A). Diet of the dam was switched between pregnancy and lactation rather than cross-fostering pups, as cross-fostering has previously been shown to have profound effects on offspring cardiovascular and metabolic phenotypes (23). As observed previously (Figure 2B), maternal weight did not differ significantly for any of the groups during the pregnancy period, whereas dams on HFD during pregnancy and lactation and dams on HFD during lactation only, lost weight during the initial lactation period (data not shown).

As demonstrated in our initial studies, none of the male offspring of any groups had a body weight phenotype within a week of weaning (data not shown), whereas the weight of female offHFD/HFD remained increased for a brief period after weaning (Figure 7B). Female offspring of mothers that had received HFD specifically during lactation (offC/HFD) also initially had increased body weight. In contrast, offspring of dams fed HFD specifically during pregnancy only (offHFD/C) had no body weight phenotype at any stage (not even at weaning) and were identical to controls (offC/C) (Figure 7B). These data suggest that the initially increased body weight observed in female offspring of pregnancy+lactation HFD-fed mothers is largely due to processes occurring during the postnatal lactation period.

Our previous data suggested that maternal diet ultimately misprograms offspring maintenance of glucose homeostasis. As in our initial studies, during a 6-hour fast 8-week-old female offHFD/HFD remained hyperglycemic despite normal body weight (Figure 7C). Interestingly, female offspring of mothers that had received HFD during pregnancy (offHFD/C) also had fasting hyperglycemia, whereas maternal HFD-feeding during lactation (offC/HFD) had no effect on offspring blood glucose responses to the fast (Figure 7C). Male offspring had no phenotype, as shown previously (data not shown). These data demonstrate that the in utero environment is the critical period for appropriate programming of offspring glucose homeostasis maintenance.

## Discussion

These data identify a striking difference in male versus female PVH gene expression responses to glycemic state and maternal diet, along with female offspring of HFD-fed dams being particularly vulnerable to metabolic disturbance. We further identify the developmentally critical time periods during which maternal diet influences aspects of offspring metabolic balance; whereas increased weight gain is programmed by postnatal processes, disturbances in the maintenance of offspring glucose homeostasis are a consequence of in utero effects.

### Offspring metabolic alteration is programmed by maternal diet

An important and novel aspect of the maternal dietary programming model used in this study is that dams fed a HFD during pregnancy and lactation do not have increased weight, glucose levels, adiposity, or altered insulin tolerance; thus observed offspring effects are due to the maternal diet, not maternal obesity. Although a wealth of data reports offspring metabolic dysregulation from obese mothers of multiple species (5–7), few determine whether effects are due to maternal obesity or maternal diet during pregnancy and or lactation and even fewer investigate both offspring sexes.

Other groups have reported that maternal obesity is necessary for the programming effects of maternal HFD consumption on offspring, however only male offspring were examined such that any effects on females may have been missed (24). In line with our findings, primate data concluded that maternal HFD-feeding caused offspring metabolic perturbation independent of maternal obesity downstream of HFD-mediated placental dysfunction (6, 12). HFD-mediated placental dysfunction could indeed be a potential explanation for in utero environmental alterations causing offspring metabolic modulation observed in our model. Another frequently discussed mechanism for offspring metabolic dysfunction is gestational overnutrition leading to fetal hyperinsulinemia and overgrowth (25, 26). Our data demonstrate that HFD-exposure during gestation leads to perturbed adult offspring glucose homeostasis, likely downstream of altered body composition, but does not result in overgrowth as such in adulthood.

### Whereas increased offspring weight gain is due to postnatal processes, dysregulation of glucose homeostasis is a consequence of in utero programming

Dams fed a HFD during pregnancy do not have significant metabolic disturbance and offHFD/C mice have no body weight phenotype at any stage. However, the offspring of dams fed a HFD during lactation (offC/HFD and offHFD/HFD) have initially increased body weight. Our data thus suggest that offspring body weight differences are programmed during the postnatal period, which is consistent with other reports (27–29). Although an effort was made to limit offspring consuming maternal HFD by weaning promptly at P21, we cannot discount the possibility that some of the effects on body weight seen in offspring of mothers fed a HFD during lactation are due to the offspring themselves eating the maternal diet. Importantly, although maternal HFD-feeding during lactation causes initially increased body weight, it does not affect adult offspring glucose homeostasis.

In contrast, female offspring of dams fed a HFD specifically during pregnancy have glucose homeostasis dysregulation despite normal weight, uncovering that the pathways regulating offspring maintenance of glucose homeostasis are programmed by maternal diet in utero. Glucose homeostasis dysregulation in female offspring of HFD-fed dams is likely to be a consequence of their increased adiposity; nevertheless, this does not take away from the fact that exposure to maternal HFD during the lactational period does not lead to offspring glucose homeostasis dysregulation and thus argues that the developmental time point of HFD exposure is crucial in determining which offspring metabolic aspects are affected.

The separation of physiological consequences depending on developmental time of HFD-exposure thus suggests that the pathways regulating maintenance of body weight or ultimately glucose homeostasis, be it downstream of adiposity, develop at different times. Previous data have shown that maternal obesity and hyperglycemia affect offspring neurodevelopment; hypothalamic neurocircuits from the ARC to the PVH are underdeveloped in offspring of obese or gestational diabetic dams and this may be due to a prolonged postnatal leptin surge (10, 30, 31). Indeed, studies have demonstrated that leptin's neurotrophic action affecting hypothalamic connectivity is important during the neonatal period, specifically P4–P12 (32). Exposure to other hormones involved in the maintenance of body weight homeostasis, for example, ghrelin, affects offspring in a developmental-time sensitive manner (10, 33).

Recent data suggest that maternal HFD feeding during lactation affects hypothalamic neurodevelopment ultimately leading to glucose intolerance, and that hypothalamic insulin signaling contributes to this effect (29). However, our data suggest that pathways ultimately leading to offspring glucose homeostasis dysregulation are programmed by maternal diet in utero, as maternal HFD feeding during the lactational period did not lead to offspring glucose homeostasis perturbation. The main contributing factor to this apparent difference in offspring responses might be the actual composition of the maternal diet; our study used a high-fat (coconut oil)/high-sucrose diet, whereas Vogt et al (29) fed a high-fat (lard) diet without added sucrose. These data suggest that the mechanisms determining offspring metabolic health are exquisitely sensitive to composition of maternal diet and warrant further direct comparison studies. Ultimately it is this level of detail that will be necessary to inform human pregnancy dietary advice to minimize offspring health risk.

### Female offspring of HFD-fed dams is particularly vulnerable to developing metabolic perturbation

Although both male and female offspring of HFD-fed dams are overweight and hyperglycemic at weaning, only females maintain a metabolic phenotype beyond weaning. Of note, offspring were studied in young adulthood; it is possible that other metabolic dysfunctions may emerge later in life. Several models of maternal developmental programming recognize sexual differences in offspring phenotype [for review see Aiken and Ozanne (13)] with sex affected depending on the insult and the developmental time of the insult.

Maybe most relevantly, a recent study has demonstrated female offspring to be particularly vulnerable to maternal sucrose diet (34). Although the models of developmental programming are different, a commonality is increased maternal dietary sucrose during pregnancy, arguing that female offspring may be particularly vulnerable to maternal sucrose in utero. In terms of sex-specific effects the findings of our study differ from other reports on offspring effects of maternal HFD feeding specifically during gestation and/or lactation (27, 29, 35), all of whom report a body weight phenotype in adult male offspring exposed to maternal HFD during lactation. Interestingly, all of these groups used high-fat (lard) diets without added sucrose, rather than the high-fat (coconut oil) with added high sucrose diet used in our study. This disparity in sex-specific offspring phenotype depending on composition of maternal diet further underlines not only the apparent female offspring vulnerability to maternal high sucrose diet, but also sexually dimorphic programming of the fetus depending on the exact dietary perinatal environment.

Many mechanisms for sexual dimorphism in developmental programming have been explored, including epigenetic mechanisms (36). Epigenetic mechanisms regulated by environmental changes in utero can have significant long-term effects on physiology in adulthood by permanently altering gene expression. Because many of these mechanisms are involved in for example X-chromosome silencing (37) and details of promoter methylation processes (among others) have been shown to be sexually dimorphic (38) it is conceivable that an identical in utero environment may have differential effects on epigenetically-mediated gene expression in male and female offspring. We do indeed observe sexually dimorphic PVH gene expression in male versus female offspring.

Alternatively, mechanisms might exist that alter neurocircuit development in a sexually dimorphic manner; sex steroids for example might render females more vulnerable to changes in hypothalamic connectivity development. Sex differences in CNS responses to leptin and insulin have been reported (39, 40); given the important neurotrophic actions of leptin and insulin during development, sexual dimorphism in hormone sensitivity could cause altered hypothalamic neurocircuit development in male and female offspring exposed to the same in utero environment. Offspring neurocircuit development has thus far only been investigated in males.

### Glucose- and maternal diet-sensitive PVH gene expression regulation is sexually dimorphic

The expression of select genes known to be involved in hypothalamic glucose-sensing mechanisms is significantly altered by both hypoglycemia and maternal diet in adult male CD1 mice. Modulation of male PVH gene expression in response to hypoglycemia is rapid and apparent within 60 minutes of insulin injection; interestingly this is within the time frame of previously observed glucose-sensitive AMPK- and CREB coactivator-mediated transcriptome changes (18). Although we cannot formally exclude the possibility that insulin signaling (rather than glycemic state) might contribute to regulation of PVH gene expression, we have previously demonstrated that, for example, *Irs2* mRNA expression is glucose-sensitive per se in primary hypothalamic cultures (18). To conclusively delineate between the two pathways further experiments using intracerebroventricular administration of 2-deoxyglucose as a means of inducing neuronal glucopenia would be necessary.

Strikingly, female mice show no PVH gene expression adjustment in response to hypoglycemia. In contrast to male mice, no correlation between blood glucose levels and PVH gene expression was noted in female mice in these candidate genes. We cannot rule out that other sets of PVH genes do alter with glycemic state in female mice, but nevertheless, such stark sexual dimorphism in either gene expression adjustment or gene set usage to glycemic state has not, to our knowledge, been reported previously. Whether lack of down-regulation below baseline may be partially due to lower absolute female candidate expression levels, will need to be addressed further in detail. A previous study reported glucose-sensitive modulation of *Mct2*, *Glut3,* and *Sur1* expression in lateral and ventromedial hypothalamic areas of male rats (41). The same gene expression changes were also investigated in ovariectomized and estradiol-replaced female rats and showed that estradiol treatment blunted hypoglycemia-associated gene expression changes in ovariectomized rats (42). These data thus further support the hypothesis that sex steroids have a significant effect on mechanisms conveying neuronal glucose-sensitivity.

Maternal diet-responsive sexual dimorphism in PVH gene expression is apparent from weaning and maintained into adulthood; whereas male PVH gene expression undergoes modulation in response to maternal diet, no such alteration is seen in adult females. As male offspring of HFD-fed dams has normal body weight and glucose homeostasis, it is tempting to speculate that plastic PVH transcriptome modulation is necessary to maintain normal physiology in the face of maternal HFD-feeding. However, whether sex differences in PVH gene expression in response to maternal diet are cause or consequence of the metabolic modulation remains to be proven.

Furthermore, it is certainly feasible that altered synaptic input (eg, from the ARC) might alter the PVH transcriptome and thereby PVH responsiveness to for example glycemic state. It has recently been demonstrated that maternal HFD-feeding during the lactation period affects offspring neurocircuit development and results in reduced ARC – PVH POMC neuron projections (29). However, maternal dietary programming effects on neurocircuit development have so far only been analyzed in male offspring. Previous data from small litter offspring in rats suggests that PVH neuron electrophysiological responsiveness to feeding-related peptides is altered in postnatally overfed offspring (43, 44). It will be interesting to assess whether offspring PVH neuronal electrophysiological responses to glycemic state are altered by maternal diet, be that downstream of a misprogrammed PVH transcriptome and/or misprogrammed ARC synaptic input to the PVH.

The only PVH gene that showed glucose-sensitive regulation in female offspring from HFD-fed dams was *Crh*. However, this response was also highly sexually dimorphic, with female offHFD mice showing significant up-regulation of *Crh* in response to hypoglycemia. Stress-responses have been reported to be highly sexually dimorphic (45–47). Exaggerated, stress-related PVH *Crh* gene expression in response to maternal diet may underpin increased female offspring anxiety, as reported in primate offspring of HFD-fed mothers (48).

In summary, we uncover female-specific, maternal diet-mediated in utero misprogramming of offspring metabolic balance with a striking sexual dimorphism in glucose- and maternal diet-sensitive PVH gene expression adjustment. Ultimately, increased female vulnerability to develop metabolic disturbance in response to maternal diet will propagate a vicious cycle of obesity and type 2 diabetes in subsequent generations. Further studies to assess sex-specific offspring effects and programming mechanisms of maternal dietary composition are thus warranted and urgently needed.

## References

[1] Scuteri A Sanna S Chen WM Genome-wide association scan shows genetic variants in the FTO gene are associated with obesity-related traits. PLoS Genet. 2007;3:e115 17658951 10.1371/journal.pgen.0030115 PMC1934391

[2] Loos RJ Lindgren CM Li S Common variants near MC4R are associated with fat mass, weight and risk of obesity. Nat Genet. 2008;40:768–775 18454148 10.1038/ng.140 PMC2669167

[3] Thorleifsson G Walters GB Gudbjartsson DF Genome-wide association yields new sequence variants at seven loci that associate with measures of obesity. Nat Genet. 2009;41:18–24 19079260 10.1038/ng.274

[4] Willer CJ Speliotes EK Loos RJ Six new loci associated with body mass index highlight a neuronal influence on body weight regulation. Nat Genet. 2009;41:25–34 19079261 10.1038/ng.287 PMC2695662

[5] Oken E Gillman MW Fetal origins of obesity. Obes Res. 2003;11:496–506 12690076 10.1038/oby.2003.69

[6] McCurdy CE Bishop JM Williams SM Maternal high-fat diet triggers lipotoxicity in the fetal livers of nonhuman primates. J Clin Invest. 2009;119:323–335 19147984 10.1172/JCI32661 PMC2631287

[7] Guo F Jen KL High-fat feeding during pregnancy and lactation affects offspring metabolism in rats. Physiol Behav. 1995;57:681–686 7777603 10.1016/0031-9384(94)00342-4

[8] Myers MG Jr Olson DP Central nervous system control of metabolism. Nature. 2012;491:357–363 23151578 10.1038/nature11705

[9] Plagemann A Harder T Janert U Malformations of hypothalamic nuclei in hyperinsulinemic offspring of rats with gestational diabetes. Dev Neurosci. 1999;21:58–67 10077703 10.1159/000017367

[10] Steculorum SM Bouret SG Maternal diabetes compromises the organization of hypothalamic feeding circuits and impairs leptin sensitivity in offspring. Endocrinology. 2011;152:4171–4179 21862611 10.1210/en.2011-1279 PMC3199015

[11] Fahrenkrog S Harder T Stolaczyk E Cross-fostering to diabetic rat dams affects early development of mediobasal hypothalamic nuclei regulating food intake, body weight, and metabolism. J Nutr. 2004;134:648–654 14988462 10.1093/jn/134.3.648

[12] Frias AE Morgan TK Evans AE Maternal high-fat diet disturbs uteroplacental hemodynamics and increases the frequency of stillbirth in a nonhuman primate model of excess nutrition. Endocrinology. 2011;152:2456–2464 21447636 10.1210/en.2010-1332 PMC3100625

[13] Aiken CE Ozanne SE Sex differences in developmental programming models. Reproduction. 2013;145:R1–R13 23081892 10.1530/REP-11-0489

[14] Samuelsson AM Matthews PA Argenton M Diet-induced obesity in female mice leads to offspring hyperphagia, adiposity, hypertension, and insulin resistance: a novel murine model of developmental programming. Hypertension. 2008;51:383–392 18086952 10.1161/HYPERTENSIONAHA.107.101477

[15] Jones RH Ozanne SE Fetal programming of glucose-insulin metabolism. Mol Cell Endocrinol. 2009;297:4–9 18662742 10.1016/j.mce.2008.06.020

[16] Ashford ML Boden PR Treherne JM Glucose-induced excitation of hypothalamic neurones is mediated by ATP-sensitive K+ channels. Pflugers Arch. 1990;415:479–483 2315006 10.1007/BF00373626

[17] Parton LE Ye CP Coppari R Glucose sensing by POMC neurons regulates glucose homeostasis and is impaired in obesity. Nature. 2007;449:228–232 17728716 10.1038/nature06098

[18] Lerner RG Depatie C Rutter GA Screaton RA Balthasar N A role for the CREB co-activator CRTC2 in the hypothalamic mechanisms linking glucose sensing with gene regulation. EMBO Rep. 2009;10:1175–1181 19713961 10.1038/embor.2009.177 PMC2759732

[19] Minokoshi Y Alquier T Furukawa N AMP-kinase regulates food intake by responding to hormonal and nutrient signals in the hypothalamus. Nature. 2004;428:569–574 15058305 10.1038/nature02440

[20] Brady LS Smith MA Gold PW Herkenham M Altered expression of hypothalamic neuropeptide mRNAs in food-restricted and food-deprived rats. Neuroendocrinology. 1990;52:441–447 2177853 10.1159/000125626

[21] Acosta-Martínez M Levine JE Regulation of KATP channel subunit gene expression by hyperglycemia in the mediobasal hypothalamus of female rats. Am J Physiol Endocrinol Metab. 2007;292:E1801–E1807 17311891 10.1152/ajpendo.00700.2006

[22] Vannucci SJ Clark RR Koehler-Stec E Glucose transporter expression in brain: relationship to cerebral glucose utilization. Dev Neurosci. 1998;20:369–379 9778574 10.1159/000017333

[23] Matthews PA Samuelsson AM Seed P Fostering in mice induces cardiovascular and metabolic dysfunction in adulthood. J Physiol. 2011;589:3969–3981 21669973 10.1113/jphysiol.2011.212324 PMC3179996

[24] White CL Purpera MN Morrison CD Maternal obesity is necessary for programming effect of high-fat diet on offspring. Am J Physiol Regul Integr Comp Physiol. 2009;296:R1464–R1472 19244583 10.1152/ajpregu.91015.2008 PMC2689819

[25] Silverman BL Landsberg L Metzger BE Fetal hyperinsulinism in offspring of diabetic mothers. Association with the subsequent development of childhood obesity. Ann N Y Acad Sci. 1993;699:36–45 8267335 10.1111/j.1749-6632.1993.tb18835.x

[26] Jones HN Woollett LA Barbour N Prasad PD Powell TL Jansson T High-fat diet before and during pregnancy causes marked up-regulation of placental nutrient transport and fetal overgrowth in C57/BL6 mice. FASEB J. 2009;23:271–278 18827021 10.1096/fj.08-116889 PMC2626621

[27] Sun B Purcell RH Terrillion CE Yan J Moran TH Tamashiro KL Maternal high-fat diet during gestation or suckling differentially affects offspring leptin sensitivity and obesity. Diabetes. 2012;61:2833–2841 22751689 10.2337/db11-0957 PMC3478561

[28] Oben JA Mouralidarane A Samuelsson AM Maternal obesity during pregnancy and lactation programs the development of offspring non-alcoholic fatty liver disease in mice. J Hepatol. 2010;52:913–920 20413174 10.1016/j.jhep.2009.12.042

[29] Vogt MC Paeger L Hess S Neonatal insulin action impairs hypothalamic neurocircuit formation in response to maternal high-fat feeding. Cell. 2014;156:495–509 24462248 10.1016/j.cell.2014.01.008 PMC4101521

[30] Kirk SL Samuelsson AM Argenton M Maternal obesity induced by diet in rats permanently influences central processes regulating food intake in offspring. PLoS One. 2009;4:e5870 19516909 10.1371/journal.pone.0005870 PMC2690656

[31] Bouret SG Gorski JN Patterson CM Chen S Levin BE Simerly RB Hypothalamic neural projections are permanently disrupted in diet-induced obese rats. Cell Metab. 2008;7:179–185 18249177 10.1016/j.cmet.2007.12.001 PMC2442478

[32] Bouret SG Draper SJ Simerly RB Trophic action of leptin on hypothalamic neurons that regulate feeding. Science. 2004;304:108–110 15064420 10.1126/science.1095004

[33] Inoue Y Nakahara K Kangawa K Murakami N Transitional change in rat fetal cell proliferation in response to ghrelin and des-acyl ghrelin during the last stage of pregnancy. Biochem Biophys Res Commun. 2010;393:455–460 20152815 10.1016/j.bbrc.2010.02.022

[34] Samuelsson AM Matthews PA Jansen E Taylor PD Poston L Sucrose feeding in mouse pregnancy leads to hypertension, and sex-linked obesity and insulin resistance in female offspring. Front Physiol. 2013;4:14 23423541 10.3389/fphys.2013.00014 PMC3575022

[35] Khalyfa A Carreras A Hakim F Cunningham JM Wang Y Gozal D Effects of late gestational high-fat diet on body weight, metabolic regulation and adipokine expression in offspring. Int J Obes (Lond). 2013;37:1481–1489 23399773 10.1038/ijo.2013.12 PMC3701742

[36] Gilbert JS Nijland MJ Sex differences in the developmental origins of hypertension and cardiorenal disease. Am J Physiol Regul Integr Comp Physiol. 2008;295:R1941–R1952 18971349 10.1152/ajpregu.90724.2008 PMC2685301

[37] Chow JC Yen Z Ziesche SM Brown CJ Silencing of the mammalian X chromosome. Annu Rev Genomics Hum Genet. 2005;6:69–92 16124854 10.1146/annurev.genom.6.080604.162350

[38] Kelly TL Trasler JM Reproductive epigenetics. Clin Genet. 2004;65:247–260 15025714 10.1111/j.0009-9163.2004.00236.x

[39] Clegg DJ Riedy CA Smith KA Benoit SC Woods SC Differential sensitivity to central leptin and insulin in male and female rats. Diabetes. 2003;52:682–687 12606509 10.2337/diabetes.52.3.682

[40] Shi H Strader AD Sorrell JE Chambers JB Woods SC Seeley RJ Sexually different actions of leptin in proopiomelanocortin neurons to regulate glucose homeostasis. Am J Physiol Endocrinol Metab. 2008;294:E630–E639 18171913 10.1152/ajpendo.00704.2007

[41] Vavaiya KV Briski KP Caudal hindbrain lactate infusion alters glucokinase, SUR1, and neuronal substrate fuel transporter gene expression in the dorsal vagal complex, lateral hypothalamic area, and ventromedial nucleus hypothalamus of hypoglycemic male rats. Brain Res. 2007;1176:62–70 17889836 10.1016/j.brainres.2007.08.010

[42] Vavaiya KV Briski KP Effects of caudal fourth ventricular lactate infusion on hypoglycemia-associated MCT2, GLUT3, GLUT4, GCK, and sulfonylurea receptor-1 gene expression in the ovariectomized female rat LHA and VMH: impact of estradiol. J Mol Neurosci. 2008;34:121–129 18084728 10.1007/s12031-007-9020-z

[43] Davidowa H Li Y Plagemann A Altered responses to orexigenic (AGRP, MCH) and anorexigenic (alpha-MSH, CART) neuropeptides of paraventricular hypothalamic neurons in early postnatally overfed rats. Eur J Neurosci. 2003;18:613–621 12911757 10.1046/j.1460-9568.2003.02789.x

[44] Davidowa H Ziska T Plagemann A GABAA receptor antagonists prevent abnormalities in leptin, insulin and amylin actions on paraventricular hypothalamic neurons of overweight rats. Eur J Neurosci. 2006;23:1248–1254 16553787 10.1111/j.1460-9568.2006.04636.x

[45] Rhodes ME Rubin RT Functional sex differences ('sexual diergism') of central nervous system cholinergic systems, vasopressin, and hypothalamic-pituitary-adrenal axis activity in mammals: a selective review. Brain Res Brain Res Rev. 1999;30:135–152 10525171 10.1016/s0165-0173(99)00011-9

[46] Bangasser DA Curtis A Reyes BA Sex differences in corticotropin-releasing factor receptor signaling and trafficking: potential role in female vulnerability to stress-related psychopathology. Mol Psychiatry. 2010;15:877, 896–904 20548297 10.1038/mp.2010.66 PMC2935505

[47] Sterrenburg L Gaszner B Boerrigter J Chronic stress induces sex-specific alterations in methylation and expression of corticotropin-releasing factor gene in the rat. PLoS One. 2011;6:e28128 22132228 10.1371/journal.pone.0028128 PMC3223222

[48] Sullivan EL Grayson B Takahashi D Chronic consumption of a high-fat diet during pregnancy causes perturbations in the serotonergic system and increased anxiety-like behavior in nonhuman primate offspring. J Neurosci. 2010;30:3826–3830 20220017 10.1523/JNEUROSCI.5560-09.2010 PMC2846411

